# Exploring factors influencing pre-service and in-service teachers´ perception of digital competencies in the Chinese region of Anhui

**DOI:** 10.1007/s10639-022-11085-6

**Published:** 2022-06-02

**Authors:** Li Yang, Fernando Martínez-Abad, Alicia García-Holgado

**Affiliations:** 1 GRIAL Research Group, University of Salamanca, Paseo de Canalejas 169, 37008 Salamanca, Spain; 2 IUCE, Computer Science Department, GRIAL Research Group, University of Salamanca, Paseo de Canalejas 169, 37008 Salamanca, Spain; 3 IUCE, Dpto. of Didactics, Organization and Research Methods, GRIAL Research Group, University of Salamanca, Paseo de Canalejas 169, 37008 Salamanca, Spain

**Keywords:** Assessment, Perception, Pre-service teachers, In-service teachers, Digital competence, China

## Abstract

The emergence of the Covid-19 pandemic has accelerated the wave of digital social transformation worldwide and pushed the “Accelerator Key” for the digital transformation of education in 2020. This transformation has also impacted in an all-around way in China. Taking Anhui province as a case study, this research explores socio-demographic factors influencing the digital competence level of pre- and in-service teachers of primary and secondary education in China. The quantitative methodological approach emphasizes the study subjects’ perception of their digital competencies in three factors: basic technology literacy, technical support learning, and technical support teaching. The study involved 250 pre-service teachers and 248 in-service teachers. The main findings are: (1) participants have good consciousness and attitudes towards using ICT in daily work, but their educational practice is weak; (2) in-service teachers have a digital competence level generally higher than pre-service teachers’, which might be their professional practice promote them to reflect on perceptions and attitudes regarding technological education; (3) for in-service teachers, there are significant differences between their digital competence level and age, years of teaching experience, educational background; (4) current ICT courses have no influencing on in-service teachers’ digital competence level, implying that current ICT training system may have problems. The study provides insights to improve pre-service teachers’ digital competence education in universities and develop well-designed in-service teachers’ ICT training courses.

## Introduction and conceptual framework

Over the past five years, the Chinese digital economy has developed prosperously. Many people solve their needs for daily life using technology, and high-tech exploration has accelerated as well. Based on the report from China Internet Network Information Center (CNNIC) (2021), until the end of 2020, China has achieved full coverage of Internet infrastructure, where the proportion of Chinese users accessing the Internet through their mobile phones reached 99.7%. Besides, the size of Internet users has grown steadily, that Internet penetration has reached 70.4%, which most users belong to the aged 20–29 (19.9%), 30–39 (20.4%), and 40–49 (18.7%). In the same year, the Internet penetration rate of minors reached 94.9%, and the proportion of underage netizens who use the Internet to study was 89.9% (Youth Rights Protection Department of the Central Committee of the Communist Youth League, 2021). During the pandemic period in early 2020, the average time spent online per netizen in China increased significantly by 30.8 h in a week. Even after the pandemic, the per capita weekly time spent online still was 26.2 h (Youth Rights Protection Department of the Central Committee of the Communist Youth League, 2021).

The emergence of the epidemic not only has accelerated the wave of digital social transformation in an all-around way in China, but it also has pushed the “Accelerator Key” for the digital transformation of education (García-Peñalvo, 2021; Huang, 2020; Yan et al., 2021; Zhu, 2020). According to the Ministry of Education of the People´s Republic of China (2021), one of their development goals in 2021 is to accelerate the high-quality development of education informatization, actively develop “Internet + Education,” and comprehensively guarantee the network security of the education system. In this case, the Ministry of Education focuses on the informatization to promote new educational facilities, research, and build a high-quality education support system. On the other hand, with the objectives of improving the principal’s information leadership, the teacher’s information teaching ability, and the training team’s information guidance ability, Opinions on the Implementation of the “National Primary and Secondary School Teachers’ Information Technology Application Ability Improvement Project 2.0” (2019) have been put forward before the pandemic.

As Ilomäki et al., (2011) mentioned, digital competence is an evolving policy-related concept, which has been used by OECD (2018), EU (2013) and UNESCO (2018) policy papers. European Commission (2018) defined digital competence involves the confidence and critical use of Information Society Technology (IST) for work, which is grounded on basic skills in ICT for the use of computers to retrieve, assess, store, produce, present, and exchange information, and to communicate and participate in collaborative networks via the Internet. DigComp frameworks (Carretero et al., 2017; Ferrari et al., 2013; Vuorikari et al., 2016) were formulated following this concept. This framework has been applied at larger scales, particularly in the context of education and training and lifelong learning, as an assessment tool of digital competence.

The terms “Teacher’s ICT competency” or “Teacher’s IT competency” Rao et al., 2019; Tang et al., 2019; Yao et al., 2019; Zhang et al., 2019; X. M. Zhang et al., 2019) have been used most frequently by the researchers or policymakers in China, which is a concept initially based on ICT Competency Framework for Teachers (UNESCO, 2011). Since the diagnostic information provided in the existing theoretical frameworks of digital competence in the Chinese environment seems insufficient or inadequate to support the current development status of IT applications in China education, Chinese scholars frequently cite and use theoretical frameworks from foreign countries or regions in recent years.

This study aims to measure pre-service and in-service teachers’ digital competence levels and explore the relationship between the influencing factors and their digital competence level using a theoretical framework validated in the Chinese context. The results of this study will yield insight to work on pre-service teachers’ digital competence education in universities and developing well-design in-service teachers’ ICT training courses. This study is conducted in an important eastern economic development region: Anhui province.

The paper has been organized in the following way: The next section is the literature review, including an overview of teachers’ digital competence in China. The third section is the study’s methods, describing participants, the instrument, data analysis methods, and the results of reliability and validity of the questionnaire. Then, the results of this study and its related discussion have been presented respectively in the fourth and fifth parts. Finally, the last section summarizes the main conclusions of the study.

## Literature review

### Status of teacher’s digital competence in China

Since 2015 teachers’ digital competence has been an important research topic in China. It is generally agreed that the informatization level of whole states is unbalanced among eastern, central, and western regions (Fan & Song, 2016; Zhao & Qian, 2018). The eastern area has a higher informatization level than the central and western areas. However, the development speed of informatization in the western and central areas is faster than in the eastern area, and the informatization level in the central area tends to catch up with the eastern area (Kuang et al., 2018). In the same way, teachers’ digital competence level in the western and central areas is generally inferior to those in eastern regions, above all, in teaching practice with ICT tools (Wang & Ren, 2020; Yang & Hu, 2019).

As Li, Wu, et al. (2016b) mentioned, the value of digital teaching facilities and teaching resources is seriously underestimated due to the lack of experience and knowledge in using advanced IT to integrate it into teaching. Primarily, teachers do not make full use of the latest resources available on the Internet to deepen students’ learning content, nor do they have the advantages of technology-based information retrieval and processing to propose more activities that promote students’ interest, participation, and depth in learning. Moreover, teachers have insufficient competence to design and organize activities based on technology for students to carry out cooperative learning in the classroom (Tang et al., 2019; Yao et al., 2019).

### Factors influencing teacher’s digital competence in China

For the last twenty years, several review studies have shown that various factors influence teachers’ use of ICT (Drent & Meelissen, 2008; Mumtaz, 2000; Spiteri & Chang Rundgren, 2020). Due to the passage of time and the development of society, the factors that affect teachers’ use of information technology (IT) are also changing. First of all, Kong & Zhao (2017) and Wang & Ren (2020) concluded that technical foundation, school system, teacher training, and environment have a significant direct or indirect impact on the teachers’ digital competence. Then, some Chinese scholars investigated influencing factors based on the technology acceptance model (TAM). Zhang et al., (2015), Xu & Hu (2017) and Li et al., (2017) reported that student interaction feedback as an external factor could directly affect teachers’ IT application behavior. On the other hand, Zhang et al., (2018) and Li et al., (2018) found that group influence, performance expectations, and convenience conditions as natural influencing factors can affect teachers’ IT application behavior, but self-efficacy is a vital factor. Other researchers indicated that age, years of teaching experience, and teaching subjects of teachers have significant differences in their level of digital competence. For instance, Li et al., (2016) reported that teachers’ age is an internal factor that significantly impacts their level of digital competence. Additionally, some researchers have been interested in the topic of teacher training for integrating technology into the teaching process (He et al., 2018; Huang et al., 2016; Li & Huang, 2018; Wu & Yang, 2016).

For pre-service teachers’ digital competence, it has also received attention in recent years (Li et al., 2019a; Wang & Wu, 2018). Concerning the research works for pre-service teachers’ digital competence, there has been an insufficient development on their digital competence. Firstly, there is still a gap in the IT hardware environment, hardware and software equipment, and independent campus network, including the deficiency of IT teachers and the lack of access to educational information resources (Zhou et al., 2016). According to Zhou et al., (2017), pre-service teachers’ digital competence is low in three issues: the willingness to apply IT to optimize teaching, the ability to design and organize applications ability, and the professional development awareness.

Previous studies have demonstrated several influencing factors for Chinese pre-service or in-service teachers’ digital competence. However, no one studied for Anhui province specifically nor compared work for these two groups. Thus, the objectives of this study are to assess and analyze Chinese pre-service and in-service teachers’ perception of digital competence and explore the relationship between socio-demographic factors (age, educational degree level, ICT courses, years of teaching experience) and their digital competence level in Anhui province. In this regard, we propose the following research questions:

1. What is the status of pre-service and in-service teachers’ perceptions of digital competency in China?
2. Which analyzed factors influence the level of digital competence of pre-service/in-service teachers? Furthermore, which are the stronger ones that can influence the level of digital competence of pre-service/in-service teachers?

## Method

This study proposed a diagnostic evaluation from a quantitative paradigm with a non-experimental-cross-sectional design. We explored relationships between the socio-demographic factors and pre-service and in-service teachers’ perceived digital competence level, explicitly examining three areas: basic technological literacy, technical support learning skills, and technical support teaching skills.

### Participants

**Fig. 1:**
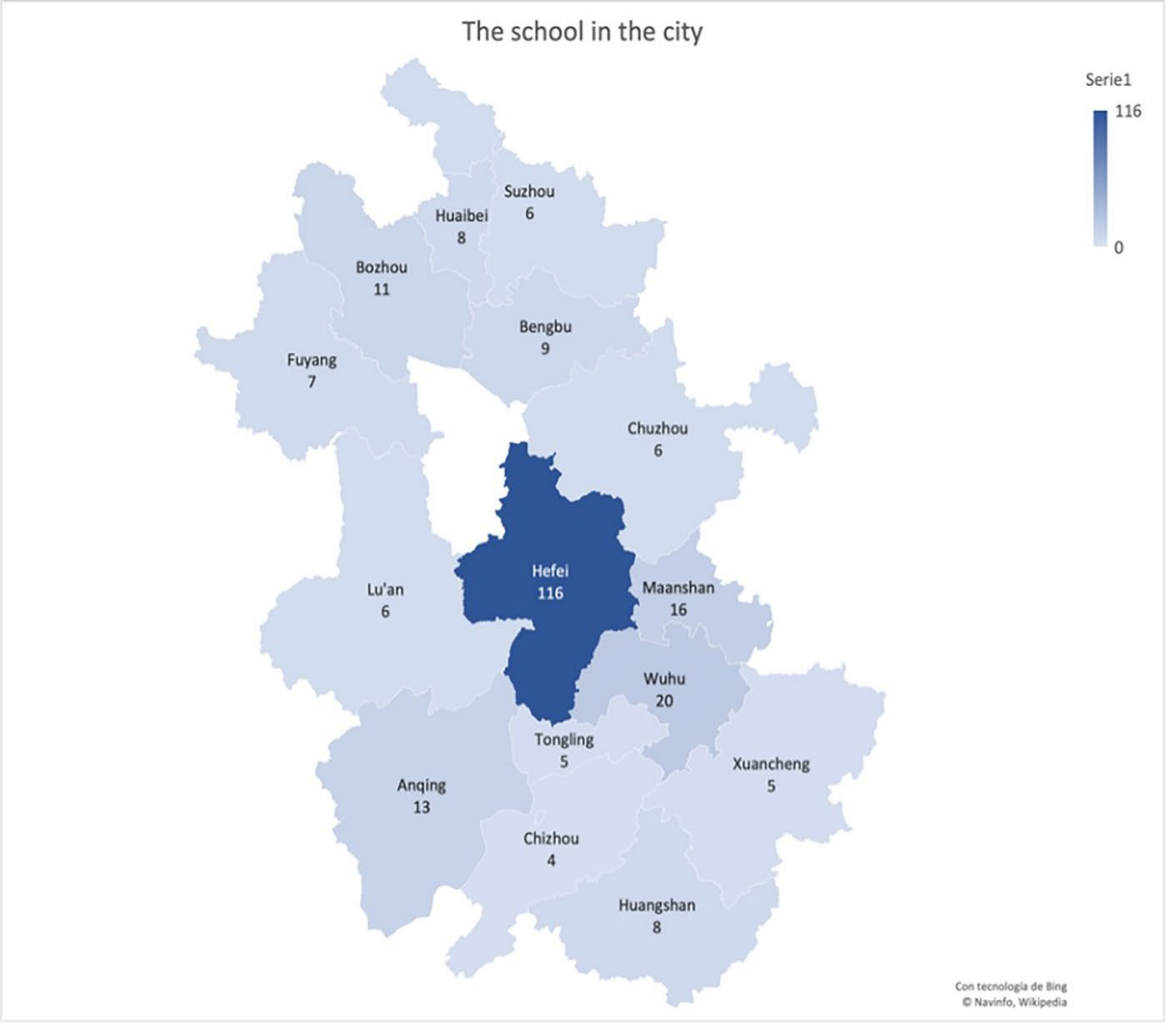
Geographical distribution of the sample

The sample was retrieved online from both pre- and in-service teachers in China’s Anhui province between February and May 2021. A non-probabilistic sampling procedure (voluntary response sample) was applied. Thus, we initially contacted via WeChat those members of the population for whom we had contact information. Finally, total of 498 answers were collected. Most participants (116) are from Hefei, the capital and largest city of Anhui Province (Fig. 1).

The sample was divided into in-service teachers (n = 248) and pre-service teachers (n = 250). For in-service teachers, there is 136 female (54.84%) and 112 male (45.16%) participants; for pre-service teachers, there is 122 female (48.8%), and 128 are male (51.2%) participants. Therefore, both groups have a balanced gender distribution.

**Fig. 2:**
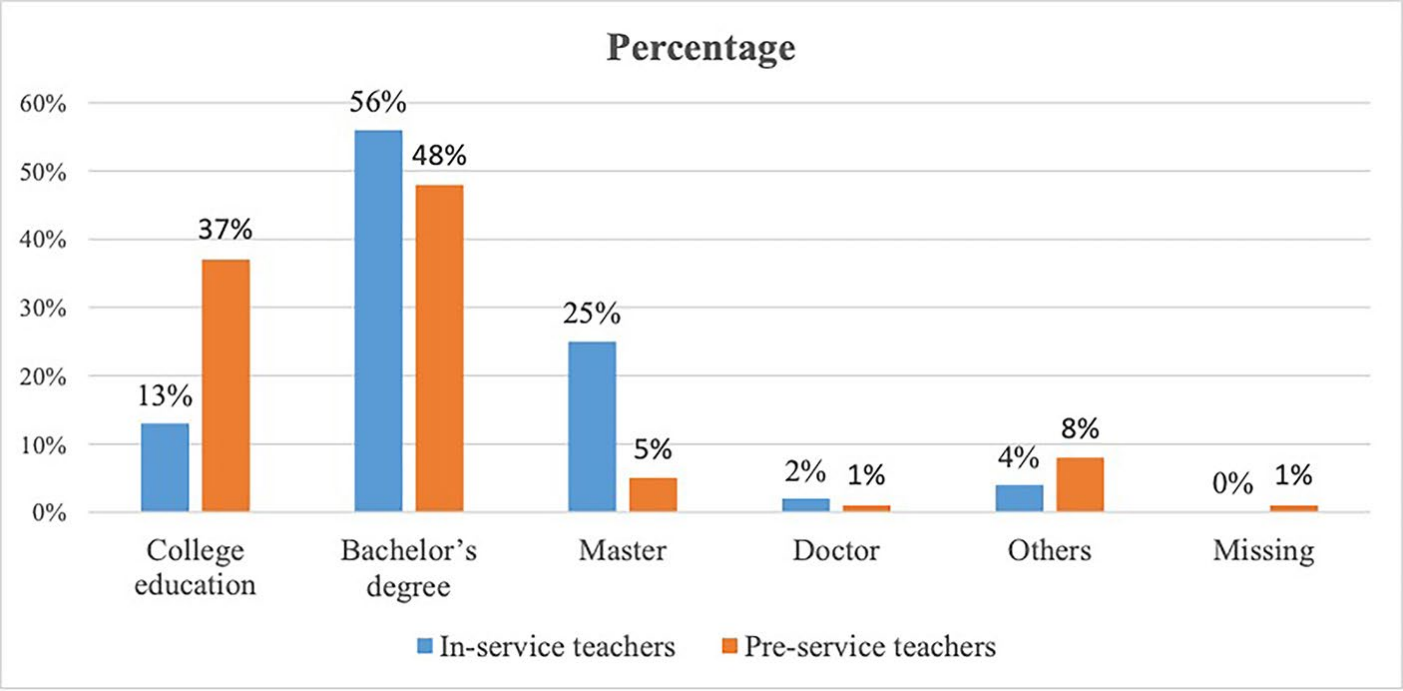
Distribution of pre-service and in-service teachers’ education background

Figure 2 shows the educational background of pre- and in-service teachers. Most participants have bachelor’s degree (56% of in-service teachers and 48% of pre-service teachers) and very few have Ph.D. (2% of in-service teachers and 1% of in-service teachers).

Table 1 shows the results of descriptive statistics of pre- and in-service teachers’ age, in which the mean of pre-service teachers’ age is 21.55 and the mean of in-service teachers’ age is 31.82. Moreover, the mean of in-service teachers’ teaching experience is 7.92 years. According to p_25_ and p_75_, half of the teachers have experienced between 3 and 10 years.

**Table 1:** Descriptive statistics of participants’ age and in-service teachers’ teaching experience

	Mean	S_X_	Min	P_25_	Mdn-P_50_	P_75_	Max
Age (Pre-service)	21.55	2.70	16	20	21	23	36
Age (In-service)	31.82	6.84	17	28	30	35	55
Teaching Exp.	7.92	7.84	0	3	5	10	34

**Fig. 3:**
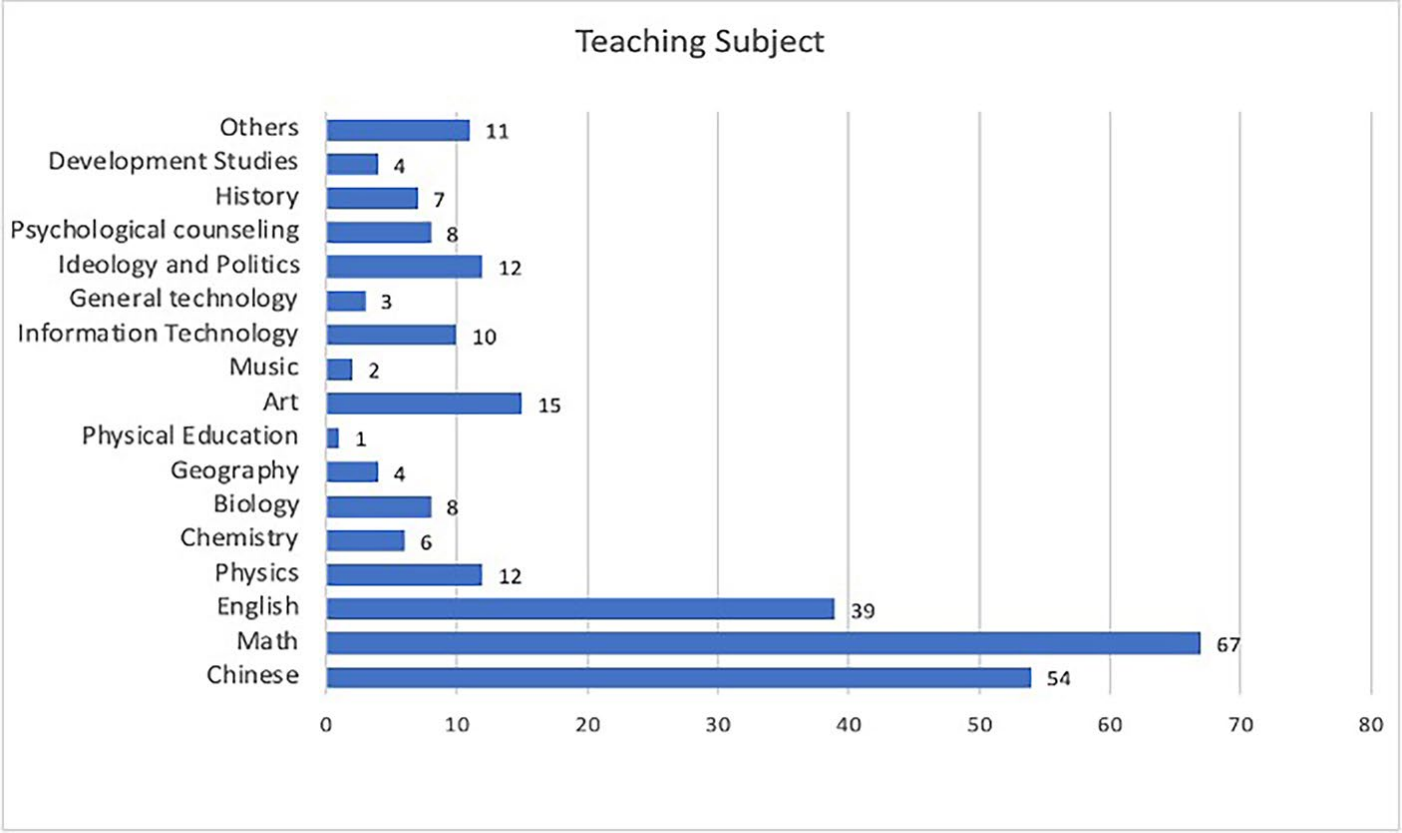
Distribution of in-service teachers’ teaching subject

Regarding in-service teachers’ job titles, 175 participants are subject teachers (68%), 31 participants are grade leaders (12%), followed by 23 research leaders (9%). It is worth noting that these job titles can overlap. Figure 3 shows the distribution of in-service teachers’ teaching subjects, which have 67 mathematics teachers (26.8%), 54 Chinese teachers (21.6%), 39 English teachers (15.6%), 15 art teachers, 12 physics teachers, and 12 teachers engaged in teaching ideology and politics subject.

### Instrument

**Fig. 4:**
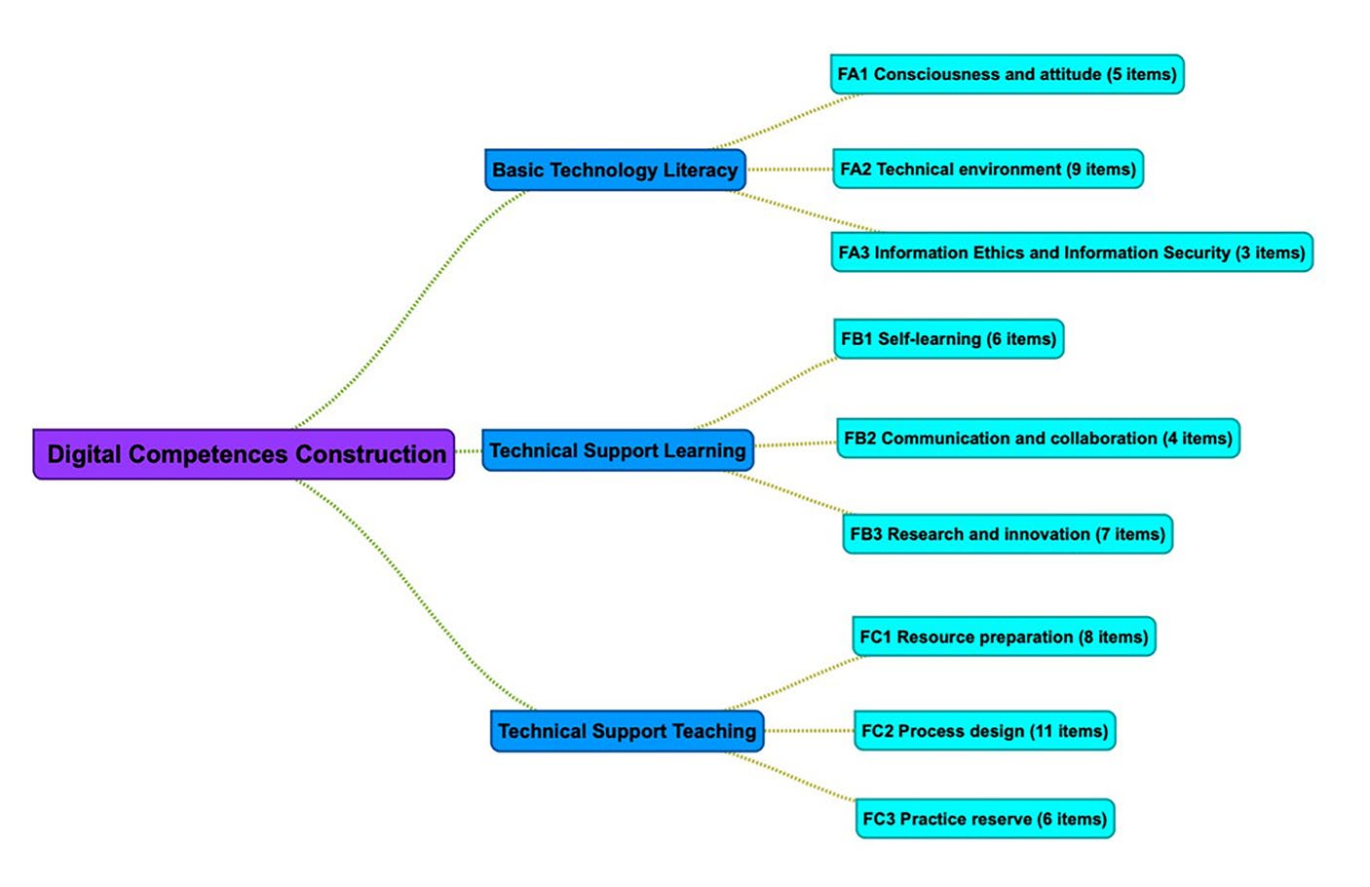
Dimensional structure of the instrument

According to the previous literature review studies, we considered using the instrument proposed by Yan et al., (2018). It has formed by three fundamental measured factors (Basic Technology Literacy, Technical Support Learning, and Technical Support Teaching), and each factor consists of three dimensions (Fig. 4). This instrument is based on the Chinese theoretical framework “Information Technology Application Ability Standards for Primary and Secondary School Teachers (Trial)” (Ministry of Education of the People´s Republic of China, 2014), and it is validated for Chinese pre-service teachers. Since there is no suitable ICT assessment tool for current pre-service teachers in China, evaluating their digital competence is challenging, and training units are challenging to improve their digital competence level. Yan et al., (2018) designed and validated this instrument to effectively diagnose pre-service teachers’ self-perceived digital competence to provide a scientific basis for pre-service teachers’ digital competence training.

Hence, the scale included in the questionnaire used in this study was translated and validated from this instrument. The questionnaire used consisted of two parts: (1) socio-demographic initial questions; and (2) sixty subjective five-level Likert response questions (strongly agree [5], agree [4], no agree, neither disagree [3], disagree [2], and strongly disagree [1]). This study sought to determine the accuracy and validity of subjective self-assessment of digital competence for study subjects through socio-demographic questions. In determining the impact of socio-demographic and experience variables, the results can point to factors influencing the instruction design and program development for pre-service and in-service teachers.

### Data collection

The online questionnaire was conducted according to institutional review board privacy and security before sending it to undergraduate students in the educational field at a large Anhui province public university. The research’s objective was explained, and the collaboration of the students (pre-service teachers) was requested by encouraging them to participate in the study. At the same time, the questionnaire was sent to in-service teachers who engaged in primary and secondary school in Anhui province. For data collection, the questionnaire was administered during free time, so its application would not interfere with the usual rhythm of the classes. Finally, the survey was completed by 625 anonymous participants, then 498 participants remained for inclusion in the study after identifying and cleaning the data from uncompleted or low credibility questionnaires.

### Data Analysis

All the data obtained for this study were analyzed by SPSS version 26 and JASP version 0.14.1. Firstly, for validating the theoretical structure of the instrument, Confirmatory Factor Analysis (CFA) techniques were used. Then, GFI, SRMR, NFI, RFI, CFI, and a chi-square test were applied for assessing the goodness of fit of the model’s well-known indices. The average variance extracted (AVE) and composite reliability (CR) for the general explained variance and the internal consistency.

Descriptive, correlational, and inferential statistics were used to analyze the socio-demographic questions, factors, and dimensions. Lastly, after applying the Shapiro-Wilk test and computing skewness and kurtosis for analyzing the normality assumption, we applied the Pearson correlation coefficient to compare scale variables and parametric (t-test or one-way ANOVA) or non-parametric (Mann-Whitney or Kruskal-Wallis) tests. The signification level of 5% has been used in all hypothesis contrasts, and the appropriate effect size statistic (Cohen’s d, eta squared or rank-biserial correlation) has been included.

### Reliability and validity of the questionnaire

For calculating reliability for each of the nine dimensions and the three factors, the Cronbach’s Alpha and CR coefficients were used to determine the internal consistency (reliability), CFA with the Diagonally Weighted Least Squares parameter estimation technique was applied to study the factorial validity of the scale.

Table 2 shows the results of measurement model fit indices in the CFA, evidencing the model fits of all three factors are good. The p-values of the chi-square tests and the ratio chi-square/degrees of freedom show a good fit. For the interpretation of fit indices, this table shows another fit measure parameter with an excellent index in three factors, in which the values of SRMR were less than 0.05, the values of GFI, NFI, RFI were greater than 0.90, and the vales of CFI were close to 1. So, the global fit of the model in three-factor dimensions was good.

**Table 2:** Statistics of several fit indices of the hypothetical model

		Value		
		FA Consciousness and attitude	FB Technical environment	FC Information Ethics and Information Security
chi^2^		78.876	26.184	113.693
df		116	116	296
p		0.997	< 0.999	< 0.999
Ratio (x^2^/df)		0.668	0.226	0.384
Absolute fit index	GFI	0.991	0.998	0.996
	SRMR	0.047	0.029	0.036
Incremental fit index	CFI	< 0.999	< 0.999	< 0.999
	NFI	0.986	0.997	0.995
	RFI	0.984	0.996	0.994

Table 3 shows that Cronbach’s alpha is greater than 0.8 throughout, indicating the reliability of the table is acceptable. Then CR and AVE for convergence validity in this study have been shown that CR is greater than 0.6 throughout. Four dimensions’ (FA2, FA3, FB2, FB3) average variance extracted (AVE) are greater than 0.4, and the factor loading reached good values (higher than 0.50), indicating that the reliability of this model is good.

**Table 3:** Results of CFA, their factor loadings, and reliabilities of the model

		Factor loadings	Cronbach’s Alpha	CR	AVE
FA1 Consciousness and Attitude	IT 1	0.597	0.818	0.760	39.16%
	IT 2	0.622			
	IT 3	0.641			
	IT 4	0.639			
	IT 5	0.629			
FA2 Technical Environment	IT 6	0.741	0.881	0.879	44.81%
	IT 7	0.628			
	IT 8	0.696			
	IT 9	0.661			
	IT 10	0.672			
	IT 11	0.576			
	IT 12	0.690			
	IT 13	0.700			
	IT 14	0.647			
FA3 Information Ethics and Information Security	IT 15	0.669	0.803	0.786	55.19%
	IT 16	0.809			
	IT 17	0.744			
FB1 Self-learning	IT 1	0.495	0.857	0.744	32.75%
	IT 2	0.552			
	IT 3	0.550			
	IT 4	0.603			
	IT 5	0.637			
	IT 6	0.586			
FB2 Communication and Collaboration	IT 7	0.635	0.846	0.729	40.20%
	IT 8	0.646			
	IT 9	0.606			
	IT 10	0.650			
FB3 Research and Innovation	IT 11	0.612	0.916	0.850	44.85%
	IT 12	0.670			
	IT 13	0.705			
	IT 14	0.662			
	IT 15	0.676			
	IT 16	0.648			
	IT 17	0.710			
FC1 Resource Preparation	IT 1	0.600	0.894	0.802	33.63%
	IT 2	0.548			
	IT 3	0.557			
	IT 4	0.589			
	IT 5	0.630			
	IT 6	0.582			
	IT 7	0.589			
	IT 8	0.539			
FC2 Process Design	IT 9	0.692	0.935	0.881	38.31%
	IT 10	0.619			
	IT 11	0.631			
	IT 12	0.621			
	IT 13	0.585			
	IT 14	0.562			
	IT 15	0.627			
	IT 16	0.639			
	IT 17	0.620			
	IT 18	0.612			
	IT 19	0.596			
	IT 20	0.615			
FC3 Practice Reserve	IT 21	0.656	0.884	0.788	38.31%
	IT 22	0.568			
	IT 23	0.628			
	IT 24	0.654			
	IT 25	0.596			
	IT 26	0.607			
Basic Technology Literacy	FA1	0.591	0.880	0.661	39.48%
	FA2	0.662			
	FA3	0.630			
Technical Support Learning	FB1	0.560	0.925	0.631	36.33%
	FB2	0.615			
	FB3	0.631			
Technical Support Teaching	FC1	0.562	0.944	0.612	34.49%
	FC2	0.605			
	FC3	0.594			

## Results

### Descriptive analysis

The following are the results obtained from the pre-service teachers and in-service teachers. They answered 60 measured questions composed of three core factors: Basic Technology Literacy (17 items), Technical Support Learning (17 items), and Technical Support Teaching (26 items). As mentioned above, to avoid bias, participants responded on a Likert-type scale of 1 to 5.

Table 4 shows the descriptive statistical results by dimensions and factors for all participants, in which the values of means, standard deviations, minimum and maximum, the P_25_, P_50_, and P_75_ percentiles have been reported.

**Table 4:** Statistical descriptive analysis

Factor	Dimension	Mean	S.D.	Min	P_25_	P_50_	P_75_	Max
Basic Technology Literacy	FA1 Consciousness and attitude	3.96	0.753	1	3.60	4.00	4.40	5
	FA2 Technical environment	3.86	0.689	1	3.56	4.00	4.22	5
	FA3 Information Ethics and Information Security	4.14	0.850	1	3.67	4.33	5.00	5
Technical Support Learning	FB1 Self-learning	3.86	0.67	1	3.50	4.00	4.27	5
	FB2 Communication and collaboration	3.88	0.72	1	3.50	4.00	4.25	5
	FB3 Research and innovation	3.82	0.71	1	3.43	4.00	4.29	5
Technical Support Teaching	FC1 Resource preparation	3.88	0.64	1	3.50	4.00	4.25	5
	FC2 Process design	3.86	0.65	1	3.50	4.00	4.25	5
	FC3 Practice reserve	3.86	0.68	1	3.50	4.00	4.33	5
	Basic Technology Literacy	3.97	0.72	1	3.65	4.08	4.44	5
	Technical Support Learning	3.85	0.66	1	3.65	4.00	4.24	5
	Technical Support Teaching	3.86	0.63	1	3.65	3.97	4.25	5

Regarding pre-service and in-service teachers’ digital competence in Basic Technology Literacy, the three dimensions mean values were 3.96, 3.86, and 4.14. For Technical Support Learning, the means of its three dimensions were 3.86, 3.88, and 3.82. For Technical Support Teaching, the means are 3.88, 3.86, and 3.86. Then, the means of these three factors are 3.97, 3.85, and 3.86, respectively, and their 25th percentile is 3.65, and the 75th percentile is 4.44, 4.24, and 4.25.

**Fig. 5:**
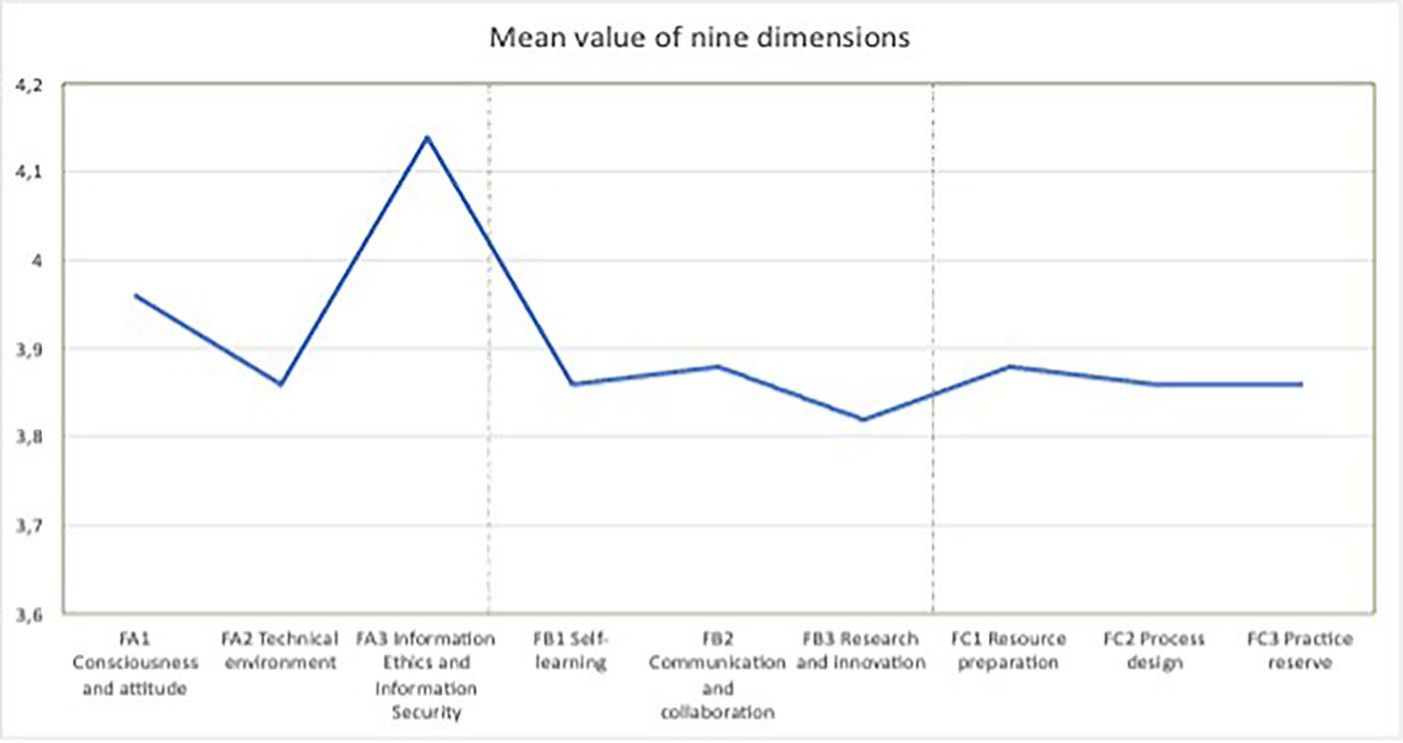
Trend chart for nine dimensions. Mean score

Based on the means of nine dimensions in Table 4; Fig. 5 demonstrates the overall trend of means is decline. As the means were all over 3.8, participants believed they had a considerable good level of basic technology literacy, technical support learning, and teaching. This figure demonstrates that the participants’ attitudes towards information ethics and security are above 4.1.

### Inferential analysis

#### Occupation

Table 5 compares the digital competence levels of pre- and in-service teachers, with significant differences in two dimensions (FA1 Consciousness and Attitude and FA2 Technical Environment). The rank-biserial correlation in these two contrasts evidence has small effect sizes of the differences, indicating that in-service teachers have more robust digital consciousness and better technical environment than pre-service teachers.

**Table 5:** Digital competence levels of in-service teachers and pre-service teachers. Mann-Whitney U test

	In-service		Pre-service		W	Sig.	r
	Mean	SD	Mean	SD			
FA1 Consciousness and attitude	4.027	0.724	3.891	0.774	52457.50	0.029	0.101
FA2 Technical environment	3.905	0.707	3.782	0.747	51782.00	0.045	0.093
FA3 Information Ethics and Information Security	4.160	0.823	4.114	8.745	48094.50	0.630	0.022
FB1 Self-learning	3.928	0.592	3.800	0,724	51100.50	0.101	0.076
FB2 Communication and collaboration	3.923	0.704	3.844	0.745	49036.00	0.320	0.046
FB3 Research and innovation	3.842	0.698	3.801	0.725	48134.00	0.568	0.026
FC1 Resource preparation	3.906	0.610	3.846	0.665	48125.50	0.482	0.033
FC2 Process design	3.870	0.638	3.853	0.666	46232.50	0.915	0.005
FC3 Practice reserve	3.875	0.633	3.849	0.713	46407.00	0.962	0.002
Basic Technology Literacy	4.032	0.680	3.915	0,752	51613.00	0.088	0.079
Technical Support Learning	3.898	0.610	3.810	0.706	49469.50	0.372	0.042
Technical Support Teaching	3.885	0.591	3.844	0.658	47748.50	0.650	0.021

**Fig. 6:**
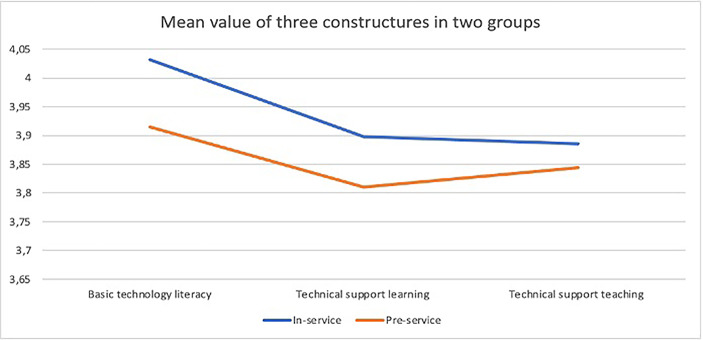
Comparing means of three measured factors in two groups

Based on the results from Table 5; Fig. 6 demonstrates ºgeneral tendency of digital competence level of participants in three areas. In-service teachers’ digital competence is better than pre-service teachers, in which both groups have the highest level in terms of Basic Technology Literacy and the lowest level in the sections of Technical Support Leaching.

#### Educational background

Regarding in-service teachers, there are significant differences between education degree levels and some areas of digital competence (Table 6). Such as FB3 Research and innovation (p = .007 < .05), FC1 Resource preparation (p-value = 0.010 < 0.05), FC2 Process design (p-value = 0.029 < 0.05), the factors Technical Support Learning (p-value = 0.30 < 0.05) and Technical Support Teaching (p-value = 0.20 < 0.05). These results indicated that in-service teachers with higher education degrees have better digital competence levels in research and innovation, resource preparation, and process design. In general, the higher education level in-service teachers have better digital competence in technical support learning and teaching.

**Table 6:** Results of Kruskal-Wallis’s test between digital competence level and in-service teacher’s educational background

In-service	Mean			K-W	Sig.	h^2^
	College	Bachelor	Master/PhD			
FA1 Consciousness and attitude	4.121	4.007	4.068	0.313	0.855	0.003
FA2 Technical environment	3.845	3.857	3.972	1.232	0.540	0.005
FA3 Information Ethics and Information Security	4.404	4.075	4.240	3.947	0.139	0.021
FB1 Self-learning	3.889	3.870	4.044	4.919	0.085	0.017
FB2 Communication and collaboration	3.939	3.844	4.051	4.228	0.121	0.017
FB3 Research and innovation	3.753	3.759	4.025	9.883	0.007	0.029
FC1 Resource preparation	3.848	3.838	4.092	9.178	0.010	0.036
FC2 Process design	3.753	3.829	3.995	7.073	0.029	0.018
FC3 Practice reserve	3.843	3.818	3.983	4.374	0.112	0.013
Basic Technology Literacy	4.123	3.980	4.093	0.995	0.069	0.008
Technical Support Learning	3.861	3.824	4.040	6.988	0.030	0.024
Technical Support Teaching	3.815	3.824	4.023	7.767	0.020	0.023

According to the results of Kruskal-Wallis’s test, Table 7 shows Dunn’s post-hoc test, which has been applied for FB3 Research and innovation, FC1 Resource preparation, FC2 Process design, Technical Support Learning, Technical Support Teaching.

**Table 7:** Dunn’s post-hoc comparisons test for several significant dimensions and factors

In-service		Mean Diff.	Z	Sig.	d
FB3 R&I	College-Bachelor	-0.006	-0.019	0.492	− 0.007
	College-Master/PhD	-0.272	-2.138	0.032	− 0.416
	Bachelor-Master/PhD	-0.266	-3.037	0.004	− 0.380
FC1 R&P	College-Bachelor	0.011	0.076	0.470	0.017
	College-Master/PhD	-0.243	-1.990	0.047	− 0.468
	Bachelor-Master/PhD	-0.254	-2.950	0.005	− 0.429
FC2 P&D	College-Bachelor	-0.077	-0.572	0.284	− 0.121
	College-Master/PhD	-0.243	-2.177	0.029	− 0.384
	Bachelor-Master/PhD	-0.166	-2.369	0.027	− 0.261
Technical Support Learning	College-Bachelor	0.036	0.382	0.351	0.057
	College-Master/PhD	-0.180	-1.486	0.137	− 0.334
	Bachelor-Master/PhD	-0.216	-2.627	0.013	− 0.365
Technical Support Teaching	College-Bachelor	-0.013	0.141	0.444	− 0.022
	College-Master/PhD	-0.208	-1.777	0.076	− 0.380
	Bachelor-Master/PhD	-0.195	-2.729	0.010	− 0.329

**Fig. 7:**
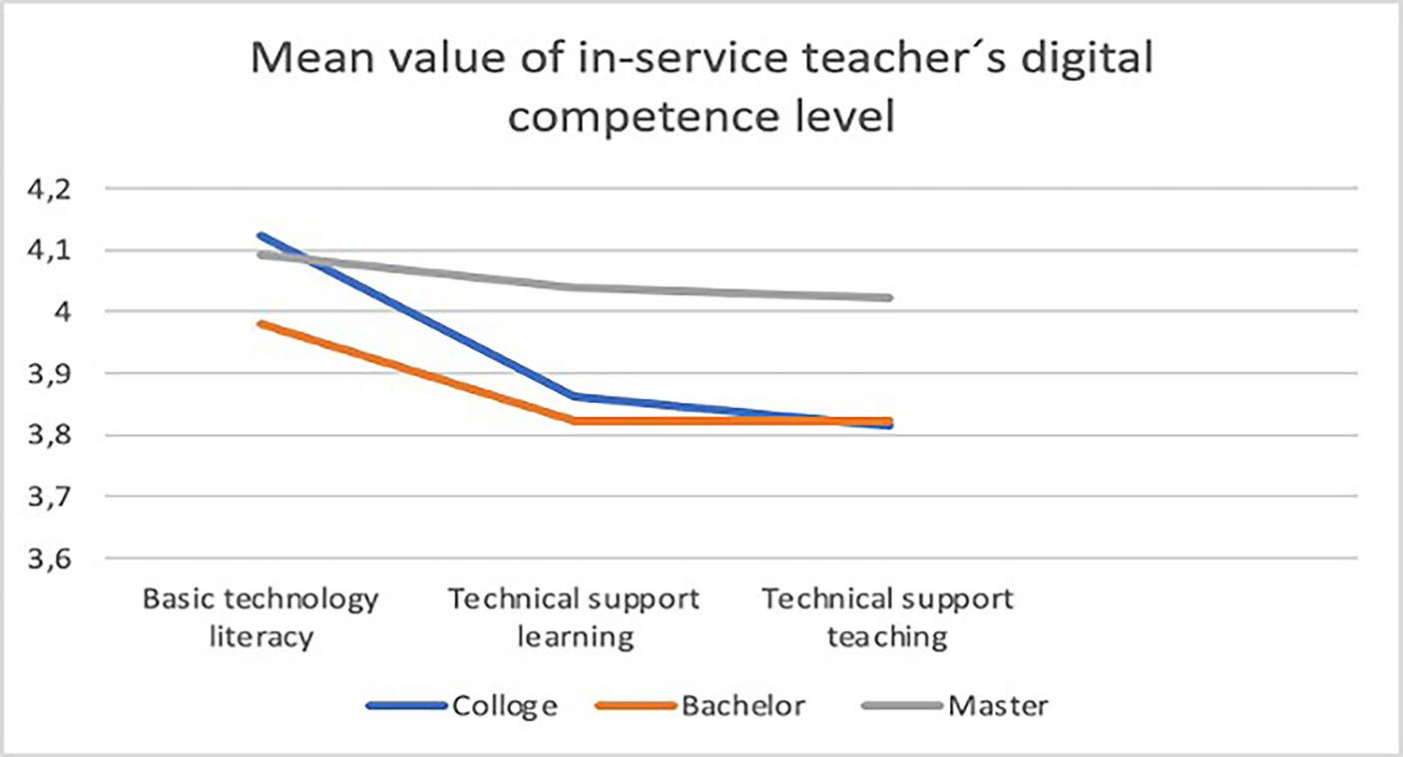
Mean scores of in-service teachers’ digital competence level in three factors with different educational backgrounds

On the other side, Fig. 7 demonstrates that teachers with master’s degrees or Ph.D. have the highest digital competence level, especially in the factor of Technical Support Learning (FB1 Self-learning, FB2 Communication and collaboration, FB3 Research and innovation), and the factor of Technical Support Teaching (FC1 Resource preparation, FC2 Process design, FC3 Practice reserve).

For pre-service teachers, there are no significant differences between any areas of digital competence and educational background, in which the p-values of each dimension are all greater than 0.05 (Table 8). Pre-service teachers’ education degree level does not influence their digital competence level.

**Table 8:** Results of Kruskal-Wallis’s test between digital competence level and pre-service teacher’s educational background

Pre-service	Mean			K-W	Sig.	h^2^
	College	Bachelor	Master/PhD			
FA1 Consciousness and attitude	4.045	3.976	3.867	2.625	0.269	0.005
FA2 Technical environment	3.940	3.838	3.800	1.638	0.441	0.006
FA3 Information Ethics and Information Security	4.254	4.168	4.467	2.130	0.345	0.009
FB1 Self-learning	2.927	3.801	3.989	2.409	0.300	0.011
FB2 Communication and collaboration	3.933	3.868	4.033	0.682	0.711	0.005
FB3 Research and innovation	3.951	3.820	3.905	1.451	0.484	0.008
FC1 Resource preparation	3.918	3.874	4.017	1.365	0.505	0.004
FC2 Process design	3.918	3.861	4.000	1.108	0.575	0.004
FC3 Practice reserve	3.878	3.891	4.056	0.974	0.614	0.004
Basic Technology Literacy	4.080	3.994	4.044	2.204	0.332	0.004
Technical Support Learning	3.937	3.830	3.976	1.581	0.456	0.008
Technical Support Teaching	3.905	3.875	4.024	1.467	0.480	0.003

#### Age (pre-service and in-service teachers) & years of teaching experience (in-service teachers)

For pre-service teachers (Table 9), there are significant differences between age and the areas of Basic Technology Literacy, Technical Support Learning, and Technical Support Teaching. Then, there are significant differences between age and dimensions of consciousness and attitude, communication and collaboration, resource preparation, process design, and practice reserve, which meet the conditions of the p-values less than 0.05 with small effect sizes. These results indicated that older pre-service teachers have a higher level of digital competence in the technical environment, awareness of information ethics and information security, self-learning, and research and innovation.

Similar results were obtained for in-service teachers (Table 9). There are significant differences between age and technical support learning and aspects of technical environment, communication and collaboration, research and innovation. These results suggested that younger in-service teachers noticed a good technical environment, and their self-perception of digital competence in communication and collaboration, research, and innovation are better than the older, as well as in technical support learning part. Secondly, Table 9 shows that there are significant differences between in-service teachers’ teaching experience and Technical Support Learning, Technical Support Teaching, as well as dimensions of technical environment, communication and collaboration, research and innovation, resource preparation, process design, practice reserve, which the p-values are less than 0.05 with small effect sizes. These results mean that teachers with more teaching experience have a lower digital competence in mentioned digital aspects.

**Table 9:** Pearson correlation analysis results

	Pre-service		In-service			
	Age				Teaching experience	
	R_xy_	Sig	R_xy_	Sig	R_xy_	Sig
FA1 Consciousness and attitude	0.168	0.003	− 0.005	0.927	− 0.088	0.173
FA2 Technical environment	0.088	0.116	− 0.117	0.044	− 0.173	0.007
FA3 Information Ethics and Information Security	0.062	0.273	0.029	0.615	− 0.040	0.537
FB1 Self-learning	0.092	0.099	− 0.051	0.385	− 0.151	0.019
FB2 Communication and collaboration	0.135	0.016	− 0.175	0.003	− 0.223	< 0.001
FB3 Research and innovation	0.094	0.094	− 0.116	0.046	− 0.224	< 0.001
FC1 Resource preparation	0.113	0.044	− 0.111	0.058	− 0.232	< 0.001
FC2 Process design	0.114	0.043	− 0.102	0.081	− 0.234	< 0.001
FC3 Practice reserve	0.133	0.019	− 0.073	0.213	− 0.193	0.003
Basic Technology Literacy	0.124	0.027	− 0.029	0.624	− 0.108	0.096
Technical Support Learning	0.115	0.039	− 0.128	0.028	− 0.222	< 0.001
Technical Support Teaching	0.112	0.046	− 0.099	0.091	− 0.236	< 0.001

#### ICT training courses

The Mann-Whitney test (Table 10) shows significant differences between pre-service teacher ICT training course and self-perception of digital competence in consciousness, attitude, and technical environment. This means that pre-service teachers believed that ICT training courses influence their consciousness, attitude, and technical environment, but it has not helped them in technical practice.

**Table 10:** Digital competence levels with an ICT training course for pre-service teachers. Mann-Whitney U test

ICT training course	YES		NO		W	Sig.	r
	Mean	SD	Mean	SD			
FA1 Consciousness and attitude	3.992	4.200	3.631	3.600	2132.500	0.019	0.384
FA2 Technical environment	3.879	4.000	3.479	3.444	2057.500	0.042	0.336
FA3 Information Ethics and Information Security	4.195	4.333	4.077	4.333	1651.000	0.660	0.072
FB1 Self-learning	3.867	4.000	3.808	3.667	1612.500	0.777	0.047
FB2 Communication and collaboration	3.910	4.000	3.692	3.750	1877.500	0.178	0.219
FB3 Research and innovation	3.885	4.000	3.593	3.429	1971.000	0.088	0.279
FC1 Resource preparation	3.899	4.000	3.654	3.750	1873.500	0.187	0.216
FC2 Process design	3.905	4.000	3.609	3.500	2010.000	0.064	0.305
FC3 Practice reserve	3.907	4.000	3.679	3.667	1896.500	0.158	0.231
Basic technology literacy	4.022	4.133	3.729	3.793	2011.500	0.064	0.306
Technical support learning	3.888	4.000	3.698	3.810	1856.000	0.214	0.205
Technical support teaching	3.904	3.986	3.647	3.653	1954.500	0.103	0.269

Table 11 shows no significant differences between in-service teachers’ ICT training programs and any aspects of digital competence, indicating that current ICT training programs have not significantly impacted in-service teachers’ digital competence.

**Table 11:** Digital competence levels with an ICT training program for in-service teachers. Mann-Whitney U test

ICT training course	YES (N = 212)		NO (N = 15)		W	Sig.	r
	Mean	SD	Mean	SD			
FA1 Consciousness and attitude	4.069	0.716	3.800	0.713	1998.000	0.095	0.257
FA2 Technical environment	3.871	0.724	3.859	0.822	1631.500	0.867	0.026
FA3 Information Ethics and Information Security	4.171	0.835	4.111	0.965	1586.000	0.988	− 0.003
FB1 Self-learning	3.939	0.581	3.700	0.807	1891.500	0.217	0.190
FB2 Communication and collaboration	3.913	0.713	3.750	0.802	1865.500	0.252	0.173
FB3 Research and innovation	3.848	0.719	3.467	0.848	2008.000	0.087	0.263
FC1 Resource preparation	3.922	0.607	3.658	0.613	2040.000	0.066	0.283
FC2 Process design	3.887	0.630	3.506	0.913	2000.000	0.095	0.258
FC3 Practice reserve	3.892	0.629	3.500	0.913	1956.000	0.133	0.230
Basic Technology Literacy	4.037	0.692	3.923	0.720	1775.000	0.453	0.116
Technical Support Learning	3.900	0.612	3.639	0.771	1973.000	0.119	0.241
Technical Support Teaching	3.900	0.590	3.555	0.748	2065.000	0.053	0.299

## Discussion

The focus of this study was not just measuring pre-service and in-service teachers’ digital competence level but also an exploration of influencing socio-demographic factors on their perceptions of digital competence in China, which focuses on a group of samples in Anhui province. Its sample can reflect the basic level of Chinese teachers’ digital competence. An instrument designed by Yan et al., (2018) that was validated for Chinese pre-service teachers has been applied in this study.

The descriptive results of this study demonstrated that both pre-service and in-service teachers have a good perception of digital competence in the areas of basic technology literacy, technical support learning, and technical support teaching. This finding is in line with the results in studies of Chen et al., (2019), Galindo-Domínguez & Bezanilla (2021) and Valtonen et al., (2021), which respectively demonstrated a similar result that Chinese pre-service and in-service teachers have a good perception of digital competence. Secondly, both groups of participants showed that they have good consciousness and attitude towards using IT for their daily work-life, in which their information ethics and security awareness were quite good. These results were in line with the findings of the earlier studies (Chen et al., 2020; Li et al., 2019b; Ma et al., 2019), but is opposite to the results of Chen, Zhou, Wang, et al. (2020) regarding information security cognition and solving skills. Thirdly, this study also suggested that Chinese pre-service and in-service teacher’s technical support practicing is not strong in the teaching and learning aspects, which replicates the findings of earlier studies in other countries (Charbonneau-Gowdy, 2015; Munyengabe et al., 2017; Ogodo et al., 2021; Valtonen et al., 2015; Wikan & Molster, 2011).

This study found that in-service teachers had higher perceived digital competence than pre-service teachers in three measured areas. For consciousness and attitude, and technical environment, in-service teachers show a significantly higher level than pre-service teachers, which Chen et al., (2019) suggested that increasing the frequency of ICT use would probably enhance teachers’ digital competence. The findings of this study show that though current university ICT course significantly predicted pre-service teachers’ perception, it did not affect their educational practice. Firstly, these results prove the governmental achievements in information construction for k-12 education. Secondly, we indicated that for in-service teachers, the frequent professional practice might promote them to reflect on attitudes regarding technological education to aid them in adjusting their digital competence, skills, and knowledge for technical teaching requirements.

Factors influencing pre- and in-service teacher’s digital competence have been investigated. Firstly, for in-service teachers, this study finds that compared with older, younger teachers have a higher digital competence level in terms of technical support learning. This result is similar to Barahona et al., (2020) and Li et al., (2016) mentioned; in-service teachers’ age significantly impacts their level of digital competence. This suggests that younger teachers generally have a higher digital competence than elderly teachers. On the other hand, this study indicates that in-service teachers with less teaching experience possess higher digital competence levels, contrasting findings from HIinojo-Lucana et al., (2019) and Pozo Sánchez et al., (2020). Secondly, Zhao et al., (2021) found that in-service teachers with higher educational background have better self-perception of the level of digital competence, which is in line with the result of this study that teachers with higher education degree have a better level of digital competence in technical support learning and teaching aspects. This implies that people with higher education may be more willing to learn and use ICT to service their professional practice.

For pre-service teachers, age affects their perception of digital competence, but there are no significant differences between their perception of digital competence and gender and educational background. The relation between age and digital competence level for pre-service indicates that older teachers have a higher perception of digital competence than younger teachers in all three factors. On the other side, this study confirms the findings of previous studies, which indicate that gender as a socio-demographic factor has no impact on in-service teachers’ perception of digital competence nor on pre-service teachers’ (Cabero Almenara, 2017; Tondeur et al., 2018). However, this finding is opposed to the results of Guillén-Gámez et al., (2021).

Ministry of Education of the People´s Republic of China (2019) promotes the development of teacher IT ability training in various regions through demonstration projects. Each in-service teacher should receive more than 50 h for 5 years, of which at least 50% should be practical application hours. Moreover, a series of governmental documents have been issued with the objectives of improving teacher’s digital competence level, such as Guidance from the Ministry of Education on strengthening the application of the “three classrooms” (Ministry of Education of the People´s Republic of China, 2020a), Guide for Online Training of Kindergarten Teachers in Primary and Secondary Schools (Ministry of Education of the People´s Republic of China, 2020b).

Previous studies in different countries indicated that pre-service teachers’ ICT training significantly impacts their future ICT use for learning processes and strengthens their instructional practice (Al-Abdullatif, 2019; Aslan & Zhu, 2016; Cabello et al., 2020; Valtonen et al., 2021). For instance, Tondeur et al., (2018) suggest that the self-perception of pre-service teachers’ digital competence has a significant impact on their future pupils’ ICT use. Since digital competence for teaching is a powerful skill for any education professional, Chinese universities commit to planning, designing, and evaluating digital competence throughout degrees.

Current Chinese teachers’ digital competence training is learning from Western countries; a series of reform-minded teaching practices have been applied. Similar to Li, Wu, et al. (2016a), this study relevant that based on the influential policy recommendation documents, the current ICT training programs have no impact on pre-service and in-service teachers’ digital competence. This indicated that the reform-minded teaching practice that mentors developed does not necessarily guarantee effective mentoring to support teachers’ IT learning and teaching reform. Therefore, further training (higher education or ICT training course) should be guided to make the most of digital tools in their professional practice. As well as Wang (2001) relevant the idea of collaboration in teaching and planning of teaching, teacher educators should pay attention to the influences of digital instructional contexts on mentoring and the kinds of learning opportunities that mentoring creates for teachers in different digital contexts. When designing mentoring programs and arranging mentoring relationships, teacher educators need to consider how to restructure school contexts and help teachers learn how to instruct students.

## Conclusions

The Chinese government has already created an excellent digital era in recent years. Until 2020, China has achieved full coverage of Internet infrastructure. Covid-19 has introduced considerable changes to the country’s economy and lifestyle, including in the educational field. Though the epidemic was rapidly controlled within two months in China, teachers’ digital competence still has achieved great attention in practice during the Covid-19 pandemic. From this perspective, our study focuses on pre-service and in-service teachers from one province to explore the influencing factors on their digital competence perception.

According to the findings of this study, Chinese pre-service and in-service teachers have a good perception of digital consciousness and attitude, particularly in the aspect of information ethics and security awareness. However, both pre-service and in-service teachers believed that their educational practice in technical support teaching and technical support learning parts is insufficient. Besides, in-service teachers demonstrated a higher perception level of digital competence in three areas than pre-service teachers. Furthermore, we also found that several factors (e.g., educational background, age, years of teaching experience, ICT training courses, etc.) influence pre-service or in-service teachers’ perception of digital competence. First, in-service teachers with higher education have a higher perception of digital competence, particularly in technical support teaching and technical support learning areas. Then, the age and years of teaching experience of in-service teachers were negatively correlated with the perception of digital competence. However, pre-service teachers’ age was positively correlated with the perception of digital competence. Therefore, this study indicated that age is a more decisive factor influencing the level of digital competence of pre-service and in-service teachers. Based on the findings of this study, we will give the insight to work on pre-service teachers’ digital competence education in universities and develop well-design teachers’ ICT training courses for in-service teachers.

This study has some limitations. For the data collection, because the sample consisted of primary and secondary education teachers in Anhui province, the results cannot simply be generalized to the whole country. Then, the study conducted an online questionnaire to gather the data, excluding the participants with a low level of digital competence who were not willing to answer the questionnaire. For the study’s findings, this study has a limit to investigate how current different training courses impact pre- and in-service teachers’ attitudes and behavioral intentions towards the use of ICT. Then, the instrument is designed for pre-service teachers that may be prone to underestimating in-service teachers’ digital competence.

This research has thrown up many questions in need of further investigation. Based on this quantitative study that has given the complexity of digital competence and its interrelated factors, other exploratory lines of a qualitative analysis could be considered to contrast these results more profoundly and comprehensively. On the other hand, a longitudinal study analyses the evolution of the in-service teachers’ level of digital teaching competence during the long training course. A longitudinal study investigating how pre-service teachers’ digital training course influences their future work can investigate the perceptions of the different subjects involved in the study.
